# CD36 deficiency affects depressive-like behaviors possibly by modifying gut microbiota and the inflammasome pathway in mice

**DOI:** 10.1038/s41398-020-01130-8

**Published:** 2021-01-05

**Authors:** Shunjie Bai, Wei Wang, Ting Wang, Juan Li, Shuxiao Zhang, Zhi Chen, Xunzhong Qi, Jianjun Chen, Ke Cheng, Peng Xie

**Affiliations:** 1 Department of Laboratory Medicine, The First Affiliated Hospital of Chongqing Medical University, Chongqing, China; 2 NHC Key Laboratory of Diagnosis and Treatment on Brain Functional Diseases, The First Affiliated Hospital of Chongqing Medical University, Chongqing, China; 3 Department of Neurology, Affiliated Hospital of Guizhou Medical University, Guiyang, China; 4 Department of Laboratory Medicine, Baoji Central Hospital, Baoji, China; 5 Department of Neurology, The First Affiliated Hospital of Chongqing Medical University, Chongqing, China; 6 Chongqing Key Laboratory of Cerebrovascular Disease Research, Chongqing, China

**Keywords:** Neuroscience, Diseases

## Abstract

Both inflammatory processes and gut microbiota have been implicated in the pathophysiology of depressive disorders. The class B scavenger receptor CD36 is involved in the cytotoxicity associated with inflammation. However, its role in depression has not yet been examined. In this study, we investigated whether CD36 affects depression by modulating the microbiota-gut-inflammasome-brain axis. We used CD36^−/−^ (knockout) mice subjected to chronic social defeat stress, and measured the expression of CD36 in these depressed mice and in patients with depression. The hippocampus of CD36^−/−^ mice was used to investigate changes in the NLRP3 inflammasome signaling pathway. The 16S rRNA gene sequence-based approach was used to compare the cecal microbial communities in CD36^−/−^ and WT mice. The CD36 deficiency in CD36^−/−^ mice alleviated chronic stress-induced depression-like behaviors. CD36 was upregulated in depressed mice as well as in depressed patients. Furthermore, the NLRP3 inflammasome signaling pathway was downregulated in the hippocampus of CD36^−/−^ mice. The Simpson Diversity Index revealed increased cecal bacterial alpha-diversity in the CD36^−/−^ mice. Among genera, *Bacteroides*, *Rikenella*, and *Alloprevotella* were significantly more abundant in the CD36^−/−^ mice, whereas *Allobaculum* was less abundant, consistent with the attenuated inflammation in the hippocampus of CD36^−/−^ mice. Our findings suggest that CD36 deficiency changes the gut microbiota composition, which in turn may impact depressive-like behaviors by affecting the inflammasome pathway.

## Introduction

Major depressive disorder (MDD) has its root in interactions between genetic and environmental risk factors, resulting in complex and multifactorial etiology. Depression has been reported to be associated with the alterations of HPA axis^1^, increased oxidative stress^2^, neurotrophic alterations^3^, and chronic inflammation^4,5^. The last decade has witnessed a growing interest in the contribution of microbiota-gut-brain axis to psychiatric disorders^6–8^. However, the definitive molecular mechanisms underlying the microbiota-gut-brain axis remain elusive.

Accumulating evidence suggests that the inflammasome is a key regulator of depression, and based on the inflammatory hypothesis of depression, some researchers have proposed the *inflammasome hypothesis* of depression^9,10^. Pro-inflammatory cytokines have been demonstrated to play a critical role in the induction of depressive symptomatology^5^. Caspase-1, an inflammatory factor, participates in the response of immune cells to both pathogen-derived and endogenous mediators through formation of the inflammasome. Caspase-1 has been demonstrated to play a role in inflammatory forms of cell death as well as protein cleavage and secretion^11^. In our previous study of caspase-1 knockout mice, we found that inhibition of the caspase-1 inflammatory pathway can improve the symptoms of depression and anxiety in mice through the gut-brain axis^7^. Our studie supports the concept of a gut microbiota-inflammasome-brain axis in which the gut microbiota exerts effects on brain function via the inflammasome signaling pathway^7,12^, thereby modulating inflammatory pathways, which in turn alter brain function and affect depressive and anxiety-like behaviors.

As a membrane of glycoprotein, CD36 has been revealed by our previous research to have correlations with anxiety behavior and neuropsychiatric disorders^13^. CD36 also functions as an endogenous negative regulator of angiogenesis by inhibiting growth factor-induced proangiogenic signals that mediate endothelial cell proliferation, migration, and tube formation and instead generating anti-angiogenic signals that lead to apoptosis^14,15^. As a class B transmembrane scavenger receptor for multiple ligands that expressed in numerous cell types, CD36 has been implicated in various diseases. Studies have shown that mice lacking CD36 could prevent atherosclerosis^16^ and thrombosis^17^ caused by diet, and showed limited inflammation and tissue infarction caused by acute cerebrovascular occlusion^18^, while might increase susceptibility to certain infections^19^. Notably, several lines of evidence point out that CD36 is involved in inflammation-associated cytotoxicity^20,21^. CD36 could activate intracellular signaling pathways that lead to proinflammatory chemokine and cytokine production by binding to oxidized low-density lipoproteins (oxLDLs)^22,23^. CD36 links the recognition of sterile ligands with priming and activation of the NLRP3 inflammasome in atherosclerosis, Alzheimer’s disease and type 2 diabetes^24^. Therefore, it is conceivable that CD36 participates in the inflammatory response and contributes to its deleterious effects. Although CD36 has been associated with the inflammasome, the role of CD36 in the gut microbiota-inflammasome-brain axis hypothesis of depression has not been studied to date.

For this purpose, we used CD36^−/−^ knockout mice to investigate whether CD36 participates to the induction of depression-like behaviors by regulating the inflammasome and the gut microbiome. We measured CD36 expression in depressed mice as well as in patients with depression. We also used the 16S ribosomal RNA (16S rRNA) gene sequence-based approach to compare the cecal microbial communities in CD36^−/−^ and WT mice, and we examined activation of the NLRP3 inflammasome pathway in the hippocampus of CD36^−/−^ mice.

## Materials and methods

### Animals and housing conditions

Male CD36^−/−^ mice were donated by Dr. Maria Febbraio, Lerner Research Institute, USA. Male CD36 chimeras were bred with C57BL/6 females, and agouti offspring were screened for the presence of the mutated CD36 gene by Southern blot hybridization. Offspring heterozygous for the mutation were interbred, and mice homozygous for the CD36 disrupted allele were identified by Southern blot hybridization^25^. The wild-type (WT) mice on a C57BL/6 genetic background (6–8 weeks old, 20–22 g) were purchased from the animal facility of Chongqing Medical University (Chongqing, China). Mice were bred and maintained in the Animal Resource Center of Chongqing Medical University. Mice were singly housed at least 2 weeks prior to experiments. Maintenance and experiments were performed under a 12/12-h light/dark cycle, at constant temperature (23 ± 2 °C) and relative humidity. Food and water were provided ad libitum. All animal experiments were approved by the Ethics Committee of Chongqing Medical University (permit number: 2017013), and all procedures were in accordance with the Guide for the Care and Use of Laboratory Animals^26^. The investigator was blinded to the group allocation during all the experiment.

### Chronic social defeat stress paradigm

Our chronic social defeat stress paradigm was implemented as described previously^27–30^. Prior to exposure to social defeat stress, the male CD1 mice (18–20 weeks old) were screened in a preliminary study for aggressive behavior towards a separate cohort of C57BL/6 mice to ensure the defeat of the intruder experimental mice. CD36^−/−^ and wild-type mice were randomly placed in the home cage of an aggressive resident CD1 mice exhibiting aggressive behavior for 5–10 min. Immediately after the period of physical contact, the intruder mice were transferred to the opposite compartment for 24 h. A clear perforated Plexiglas divider between the resident and intruder mice was used to separate the intruder and resident mice. During the 10-d defeat period, the C57BL/6 intruders were alternated daily, while resident aggressors were not removed from their home cage.

### Social interaction test

Social interaction was measured in an open field (40 cm × 40 cm × 18 cm) divided into three zones (one social interaction zone and two corner zones). Time spent in each zone was measured by analysis of a digital image recording for each subject. Each social interaction test is composed of two 150-s phases either with or without the target CD-1 mouse present in the interaction zone. The movements were recorded with a video recorder (DCR-SR45E, Sony, Japan) and analyzed with Ethovision XT 13.0 (Noldus, The Netherlands). In this test, the social interaction ratio (SI ratio) is obtained by dividing the time spent in the interaction zone when the target is present by the time when the target is absent^31^.

### Behavioral testing

#### Sucrose preference test (SPT)

The SPT was performed as previously described^13^. The preference for sucrose (%) = (sucrose amount/total amount) × 100.

#### Forced swimming test (FST)

The mice were placed individually in plexiglass cylinders (30 cm in height and 15 cm in diameter) filled with 15 cm of water (25 ± 1 °C). The last 5 min of the test session was scored for immobility.

#### Elevated plus-maze (EPM)

The apparatus consisted of two opposing open arms (30 cm × 5 cm × 0.5 cm) and two opposing enclosed arms (30 cm × 5 cm × 15 cm), which were connected by a central area (5 cm × 5 cm). The platform was elevated 50 cm above the floor. The mice were tested for 5 min. The time spent in the open arms and the distances traveled in the open arms were analyzed.

#### Open field test (OFT)

Each mouse was placed in the open field (50 cm × 50 cm × 40 cm) for 5 min. Distance traveled, rearings, number of entrances, and the percent time spent in the center (50% of the field) were measured^32^.

### Subject recruitment and isolation of peripheral blood mononuclear cells (PBMCs)

The protocols for clinical experimentation were reviewed and approved by the Ethical committee of Chongqing Medical University. All MDD subjects were recruited from the psychiatric center of the First Affiliated Hospital at Chongqing Medical University. MDD diagnosis relied on the Structured Psychiatric Interview using DSM-IV-TR criteria. Healthy control subjects were recruited from the medical examination center of the First Affiliated Hospital at Chongqing Medical University. Written informed consent was obtained from all recruited human subjects, and all procedures were performed according to the Helsinki Declaration. The detailed characteristics of these recruited subjects are shown in Table S1.

Fasting blood samples were collected into 10 mL EDTA-coated tubes. Plasma was centrifuged at 1200 × *g* for 15 min at 4 °C, then overlaid onto Ficoll-Paque Plus (GE Healthcare Bio-Sciences AB, Sweden). PBMCs were obtained as previously described^33^ and stored at −80 °C.

### Mouse brain sample preparation

After the tests, all mice were sacrificed simultaneously, and the brain tissues were quickly separated and frozen in liquid nitrogen. All tissues were stored at −80 °C before biochemical analysis.

### RT- qPCR analysis

For RT-qPCR, 1 μg RNA was used for cDNA synthesis using the PrimeScript RT reagent Kit (TAKARA, Japan). A SYBR green detection system (Roche, Germany) was used in the RT-qPCR reactions. β-actin was used to normalize the data. The 2^−ΔΔ^ CT method was used for data analysis, and the primer sequences are given in Table S2.

### Western blot validation

The brain tissue and PBMCs were lysed in RIPA buffer supplemented with protease and phosphatase inhibitors (Roche, Germany). Polyvinylidene fluoride membranes (Millipore, USA) were probed with primary antibodies against CD36 (1:4000; Abcam, catalog number: ab133625), NLRP3 (1:1000; Cell Signaling Technology, catalog number: 15101), ASC (1:1000; Cell Signaling Technology, catalog number: 67824), IL-1β (1:1000; Cell Signaling Technology, catalog number: 31202), Cleaved-IL-1β (1:1000; Cell Signaling Technology, catalog number: 83186), Caspase-1 (1:1000; Abcam, catalog number: ab207802), pro-Caspase-1 (1:1000; Abcam, catalog number: ab179515), NF-кB (1:1000; Cell Signaling Technology, catalog number: 8242), phospho-NF-кB (1:1000; Cell Signaling Technology, catalog number: 3033), CREB (1:1000; Cell Signaling Technology, catalog number: 9197), TrkB (1:1000; Cell Signaling Technology, catalog number: 4603), BDNF (1:1000; Cell Signaling Technology, catalog number: 47808) andβ-Actin (1:10,000; Abcam, catalog number: ab234437), overnight at 4 °C, and then incubated with secondary antibodies for 2 h. Signals were visualized with an ECL kit (Millipore, USA).

### Fecal sample collection and 16S rRNA gene sequencing

Briefly, cecal contents were collected and placed in 1.5 mL tubes, snap-frozen on dry ice, and stored at −80 °C. Microbial DNA was extracted from these samples using the E.Z.N.A. soil DNA Kit (Omega Bio-tek, Norcross, GA, USA) according to the manufacturer’s protocols. Following DNA extraction, cecal microbiota profiling was performed by paired-end 16S rRNA gene amplicon sequencing, based on the Illumina MiSeq platform (Illumina, San Diego, USA), according to the standard protocols provided by Majorbio Bio-Pharm Technology Co. Ltd. (Shanghai, China).

### 16S rRNA gene sequencing analysis

Raw fastq files were demultiplexed, quality-filtered with trimmomatic, and merged with FLASH according to the following criteria: (i) the reads were truncated at any site receiving an average quality score <20 over a 50-bp sliding window; (ii) primers were exactly matched, allowing 2-nucleotide mismatching, and reads containing ambiguous bases were removed; (iii) sequences with overlap longer than 10 bp were merged according to their overlap sequence.

Operational taxonomic units (OTUs) were clustered with 97% similarity cutoff using UPARSE (version 7.1, http://drive5.com/uparse/), and chimeric sequences were identified and removed using UCHIME. The taxonomy of each 16S rRNA gene sequence was analyzed by RDP Classifier algorithm (http://rdp.cme.msu.edu/) against the Silva (SSU123) 16S rRNA database using a confidence threshold of 70%. Alpha-diversity was calculated by the species richness indices (Chao) and species diversity indices (Simpson). For 16S function prediction, we standardized the OTU abundance table with PICRUSt, and then obtained the COG family information using the KEGG Ortholog information corresponding to the OTU with the Greengenes ID corresponding to each OTU. Finally, we calculated the abundance of each COG and KO.

### Statistical analysis

Data were expressed as the mean ± SEM, and analyzed using SPSS 21.0 software (SPSS, Chicago, IL, USA) and Prism 8 (GraphPad, San Diego, CA, USA) software. Statistical analyses were performed using unpaired Student’s *t*-tests and two-way analysis of variance where appropriate. Significant effects in two-way analysis of variance were followed by Bonferroni’s post hoc multiple comparison tests. *P* < 0.05 was considered statistically significant.

## Results

### Upregulated expression of CD36 in CSDS-exposed mice and depressed patients

**Fig. 1:**
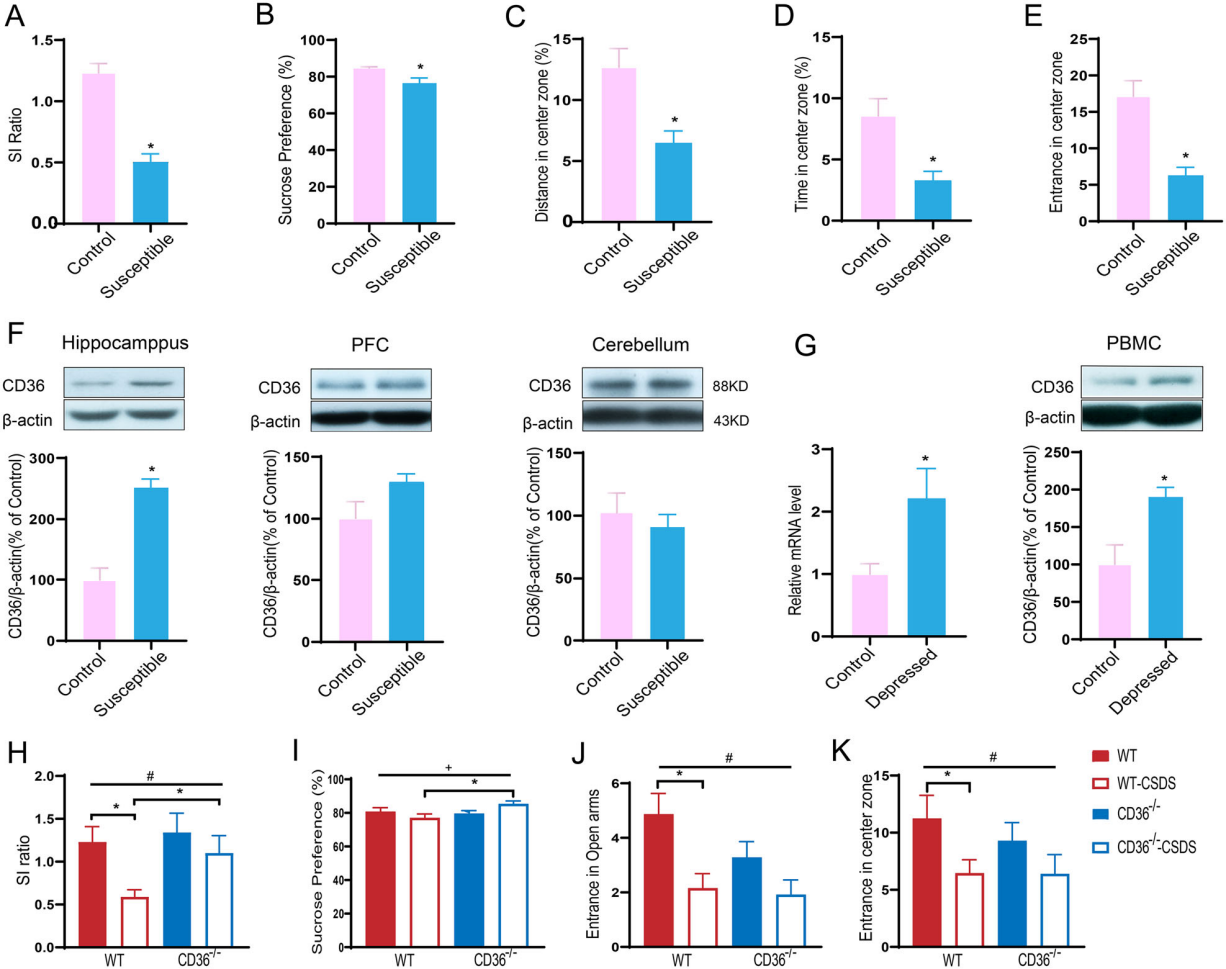
The relationship between CD36 and depression. Upregulated expression of CD36 in susceptible mice and depressed patients (a–g). **a** SI ratios for control and CSDS-exposed mice. **b** CSDS-exposed mice displayed anhedonia as measured by a reduction in sucrose preference (*n* = 15–17 mice/group, *P* < 0.05). **c**–**e** Open-field test. Compared with control mice, the distance (**c**), proportion of time (**d**), and proportion of entries (**e**) into the center were significantly decreased in the CSDS-exposed mice. **f** Representative western blot quantification of CD36 protein expression in the hippocampus, PFC and cerebellum after CSDS (*n* = 5–6 mice/group, *P* < 0.05). **g** CD36 mRNA level and representative western blotting of PBMCs in depressed patients (*n* = 16 mice/group for qPCR; *n* = 5–6 mice/group for western blotting; *P* < 0.05). Genetic ablation of CD36 in mice prevents chronic social defeat stress-induced depression-like behaviors (**h**–**k**). **h** Social interaction (SI) ratios for WT, WT-CSDS, CD36^−/−^ and CD36^−/−^-CSDS mice. CD36^−/−^ mice showed a significant increase in social interaction after CSDS. [^**#**^significant main effect of CSDS^**:**^ *F* (1, 46) = 6.419, *P* < 0.05; *P < 0.05 (post hoc Tukey test)]. **i** CD36^−/−^ mice did not exhibit anhedonia, as shown by an increase of 1% in sucrose preference after CSDS. [^**+**^significant main effect of interaction: *F* (1, 40) = 5.523, *P* < 0.05; *P < 0.05 (post hoc Tukey test)]. **j** WT mice showed decreased entrance into the open arms of the EPM. [^**#**^significant main effect of CSDS: *F* (1, 46) = 10.13, *P* = 0.093; *P < 0.05 (post hoc Tukey test)]. **k** WT mice showed decreased entry into the center of the OFT. [^**#**^significant main effect of CSDS: *F* (1, 46) = 5.114, *P* = 0.093; *P < 0.05 (post hoc Tukey test)]. WT wild-type mice, CD36^−/−^ CD36 knockout mice, CSDS chronic social defeat stress, SI ratio social interaction ratio, PFC prefrontal cortex, PBMC peripheral blood mononuclear cell.

To examine the involvement of CD36 in depression, we used the CSDS mouse model of depression. Following exposure to CSDS, the CSDS-exposed mice showed a strong tendency to spend less time in the interaction zone (Fig. 1a), and displayed a significant decrease in sucrose preference and activity in the OFT (Fig. 1b–e), compared with control mice.

Next, the levels of CD36 were examined in the CSDS-exposed mice. We found that CD36 was significantly increased in the hippocampus of CSDS-exposed mice compared with control mice (Fig. 1f). However, CD36 protein levels were not increased significantly in the PFC (prefrontal cortex) or cerebellum of CSDS-exposed mice (Fig. 1f), demonstrating regional specificity of chronic stress-induced changes in CD36. Moreover, we examined the levels of CD36 in PBMCs of patients with depression. In contrast to healthy people, depressed patients displayed a significant increase in CD36 protein and mRNA levels (Fig. 1g).

### Genetic ablation of CD36 in mice prevents chronic social defeat stress-induced depression-like behaviors

To evaluate the effect of CD36 on the depressive-like behavior induced by stress, C57BL/6J CD36 knockout (CD36^−/−^) mice were used. In our previous SHIRPA study, CD36^−/−^ mice showed a significant increase in aggressive behavior, locomotor activity, tail elevation in an open field, and anxious behavior in the OFT and EPM tests under non-stressed conditions^34^. Compared with WT mice, CD36^−/−^ mice did not display significant differences in sucrose consumption or immobility in the FST, but did exhibit a tendency towards increased sucrose consumption and decreased immobility. However, their performance under stress was not investigated.

We then compared CD36^−/−^ with WT mice in the CSDS model. Under non-defeated conditions, there were no significant difference in baseline behaviors between CD36^−/−^ with WT mice. However, in contrast to WT-CSDS mice, CD36^−/−^ mice exposed to CSDS showed increased SI and sucrose preference (Fig. 1h, i). A reduction in behavioral despair was observed in CD36^−/−^-CSDS mice, which displayed decreased immobility time in the FST (Fig. S1A). Furthermore, compared with non-stressed control mice, WT-CSDS mice displayed decreased activity in the EPM and OFT (Fig. 1j, k, and Fig. S1B). We also measured the percent alternation in CD36^−/−^ and WT mice in the Y-maze spontaneous alternation test, and no significant difference was found (Fig. S1C).

### CD36 knockout affects the gut microbiome compared with WT mice

**Fig. 2:**
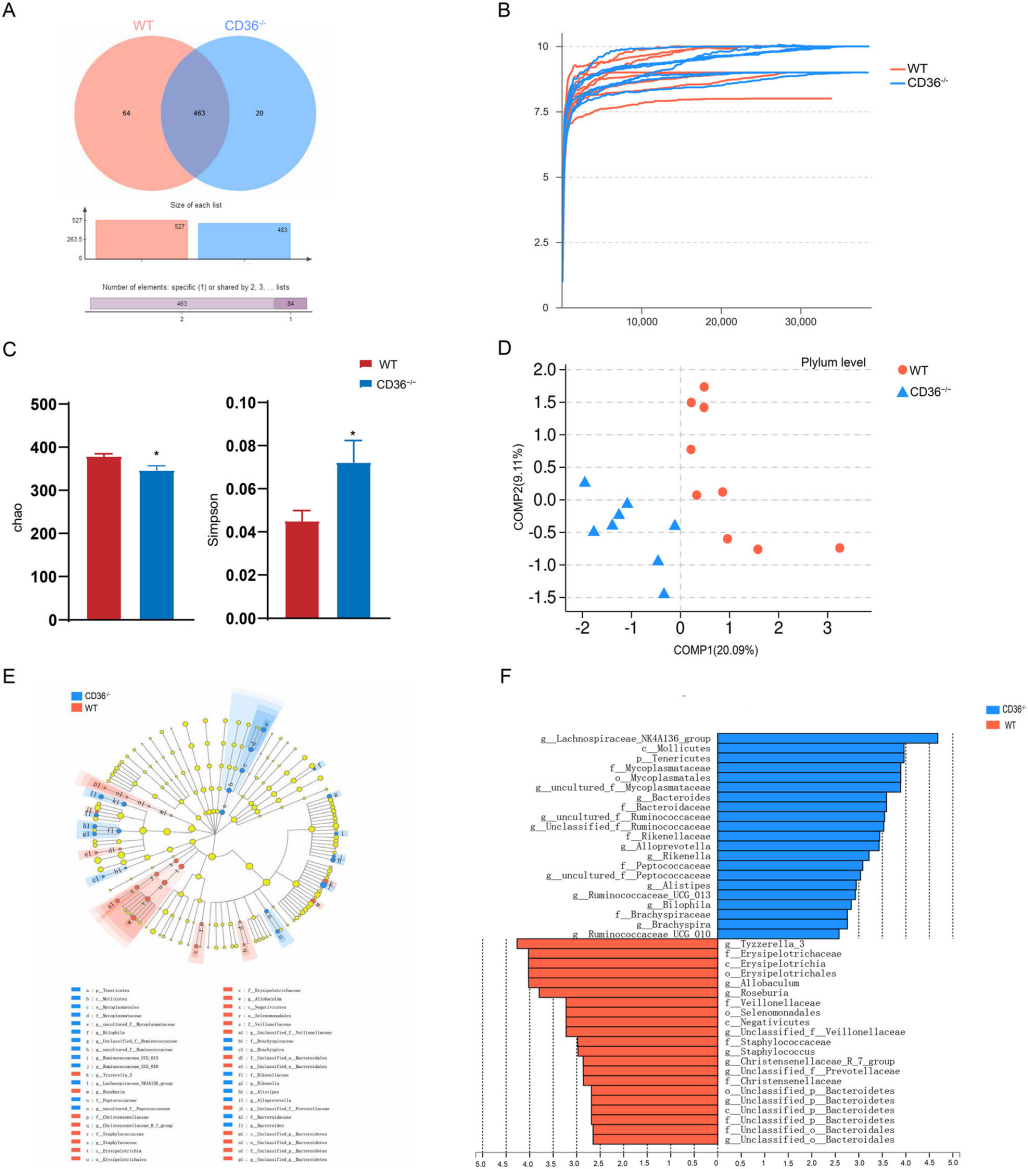
CD36 knockout affects the gut microbiome compared with WT mice. **a** A Venn diagram of OTUs detected in WT and CD36^−/−^ mice. **b** The majority of rarefaction curves tended to approach the saturation plateau. **c** Alpha-diversity analysis showed that CD36^−/−^ mice were characterized by lower microbial richness (Chao, *n* = 8–9 mice/group, *P < 0.05) and higher microbial diversity (Simpson, *n* = 8–9 mice/group, *P < 0.05) relative to WT mice. **d** At the phylum level, partial least-squares discriminant analysis (PLS-DA) showed that gut microbiota composition in CD36^−/−^ mice was greatly different from that in WT animals. **e** LEfSe identified the most differentially abundant taxons between WT and CD36^−/−^ mice—(red) WT taxa; (blue) taxa enriched in CD36^−/−^ mice. The brightness of each dot is proportional to its effect size. **f** Taxa enriched in CD36^−/−^ mice are indicated with a positive LDA score (green), and taxa enriched in WT mice have a negative score (red). Only taxa meeting an LDA significance threshold >2 are shown.

In total, we obtained 37,788 high-quality reads across all samples, with an average length of 436.11. These reads were clustered into 547 OTUs at 97% sequence similarity. Venn diagram shows that 463 of the 547 OTUs were detected in the two groups, while 64 and 20 OTUs were unique to WT and CD36^−/−^ mice, respectively (Fig. 2a). Most rarefaction curves tended to approach the saturation plateau, suggesting that the sequencing depth was enough to cover the entire bacterial diversity (Fig. 2b). Within-sample phylogenetic diversity analysis showed that the microbial richness index (Chao) was decreased and that the diversity index (Simpson) was increased in CD36^−/−^ mice compared with WT mice (Fig. 2c). These findings suggest that the microbial compositions in CD36^−/−^ mice are characterized by lower within-sample diversity. To determine whether the microbial composition in CD36^−/−^ mice was substantially different from that in WT mice, we carried out diversity analysis, and found obvious differences in gut microbial composition between the two groups from the phylum to OTU levels (Fig. 2d and Fig. S2A–F).

**Fig. 3:**
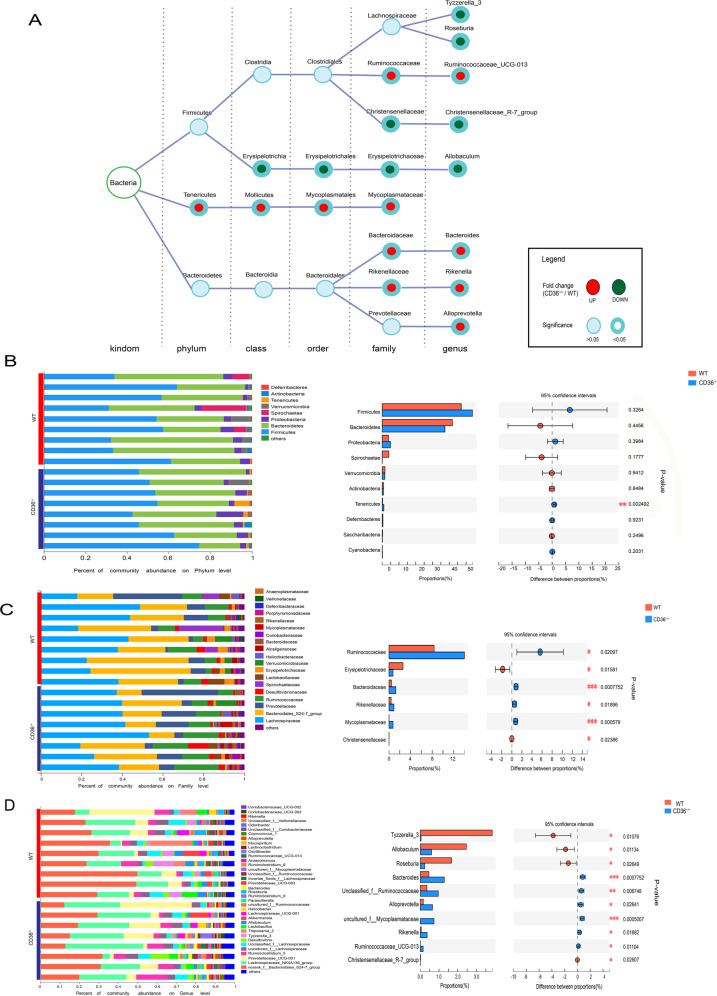
Taxonomic differences in cecal microbiota between WT and CD36^−/−^ mice. **a** The differences in taxonomic abundance between the different groups. **b** Classification and abundance of cecal contents at the phylum level. **c** Classification and abundance of cecal contents at the family level. **d** Classification and abundance of cecal contents at the genus level (*n* = 8–9 mice/group, *P < 0.05).

To further clarify the gut microbiota differences between CD36^−/−^ and WT mice, we performed Linear discriminant analysis Effect Size (LEfSe; Fig. 2e, f). The LEfSe used linear discriminant analysis (LDA) to estimate the effect of each species abundance on the difference effect. Based on LEfSe, a branch map of the gut microbiota differences between WT and CD36^−/−^ mice was showed in Fig. 2e. The predominant flora that meets the LEfSe program biomarker screening criteria (LDA score threshold >2) was showed in Fig. 2f. The LEfSe analysis revealed the differences in taxonomic abundance among the different groups (Fig. 3a). The two most abundant phylum in both groups were Firmicutes and Bacteroides, and the abundance of the phylum Tenericutes was increased sharply in CD36^−/−^ mice (Fig. 3b and Table S3). The abundance levels of the families Ruminococcaceae, Erysipelotrichaceae, Bacteroidaceae, Mycoplasmataceae, Rikenellaceae, and Christensenellaceae were also altered significantly (Fig. 3c and Table S4). Moreover, compared with WT mice, the CD36^−/−^ mice showed enrichment of the genera Bacteroides, Alloprevotella, Rikenella, and Ruminococcaceae_UCG-013, and a reduction of Tyzzerella_3, Allobaculum, Roseburia and Christensenellaceae_R-7_group (Fig. 3d and Table S5).

**Fig. 4:**
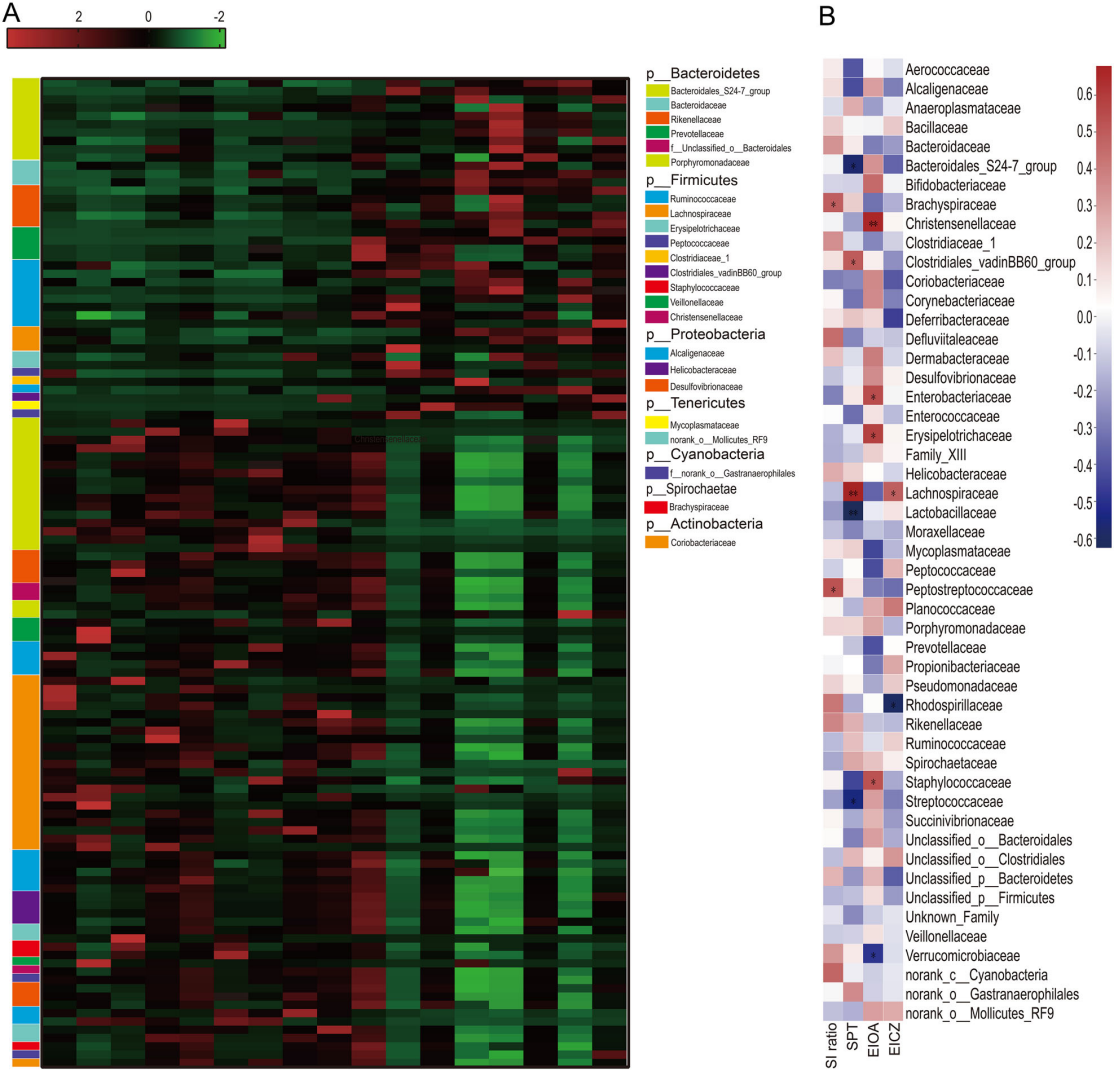
The most differentially abundant taxa between the two groups. **a** Heatmap of the 119 discriminative OTU abundances between WT and CD36^−/−^ mice (LDA > 2.0). OTUs (raw) were sorted by taxa and enrichment. The intensity of the color (green to red) indicates the score normalized abundance for each OTU. **b** Associations of gut microbial family with behavioral phenotype. Heat map of Spearman’s rank correlation. Red squares indicate positive associations between microbial family and behavioral phenotype; blue squares indicate negative associations. The statistical significance level is indicated in the squares (*n* = 8–9 mice/group, *P < 0.05). SPT sucrose preference test, EIOA entrance in open arms, EICZ entrance in center zone.

The LEfSe analysis identified 119 differential OTUs that were responsible for discriminating CD36^−/−^ mice from WT mice (Fig. 4a). To further explore the relationships between disturbances in the gut microbiome and behavioral phenotype, correlation analysis was performed. We found that the differential bacterial families were generally associated with differential behavioral phenotypes, with 24% (12/50) of bacterial families showing significant correlations with a range of behavioral phenotype (*r* > ±0.35, *P* < 0.05) (Fig. 4b).

**Fig. 5:**
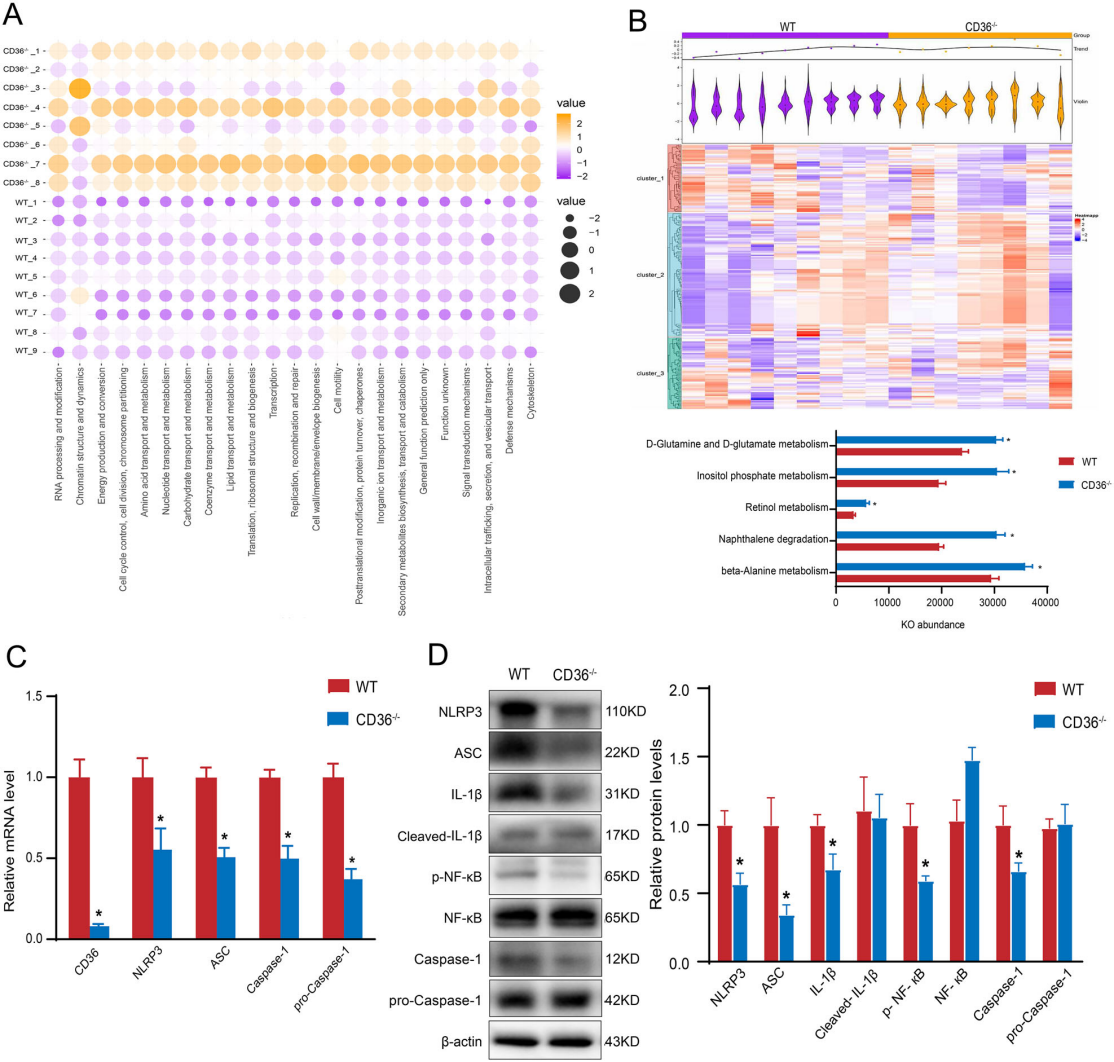
Genetic ablation of CD36 in mice alters the gut microbiota functional and the NLRP3/caspase-1 inflammatory pathway. **a** Function prediction analysis by COG functional classification. **b** KEGG pathway enrichment of gut microbiota between the two groups (*n* = 8–9 mice/group, *P < 0.05). Genetic ablation of CD36 in mice alters the NLRP3/caspase-1 inflammatory pathway (**c**, **d**). **c** Hippocampal mRNA levels of NLRP3, ASC, pro-caspase-1 and caspase-1 in CD36^−/−^ mice (*n* = 6–7 mice/group, *P* < 0.05). **d** Representative western blotting for hippocampal NLRP3, ASC, IL-1β, Cleaved-IL-1β, NF-кB, p-NF-кB, caspase-1 and pro-caspase-1 in CD36^−/−^ mice (*n* = 5–7 mice/group, *P* < 0.05).

The matrix bubble chart shows the enhanced COG functions in CD36^−/−^ mice (Fig. 5a). The COG information is presented in Supplementary 2. The KEGG pathway analysis showed that beta-Alanine metabolism, naphthalene degradation, retinol metabolism, inositol phosphate metabolism, and d-Glutamine and d-glutamate metabolism were altered in the gut microbiota metabolism pathways of CD36^−/−^ mice (Fig. 5b).

### Genetic ablation of CD36 in mice alters the NLRP3/caspase-1 inflammatory pathway

To explore the role of CD36 in the gut microbiota-inflammasome-brain axis, we measured components of the NLRP3 inflammasome signaling pathway. We observed reduced expression of NLRP3 and ASC mRNAs in the hippocampus of CD36^−/−^ mice (Fig. 5c). Furthermore, CD36 knockout also resulted in reduced expression of caspase-1 and pro-caspase-1 mRNA (Fig. 5c). As shown by western blotting, NLRP3 and ASC were decreased in the hippocampus of CD36^−/−^ mice. Decreased activation of NF-кB, IL-1β, and caspase-1 were found in CD36^−/−^ mice compared with WT mice (Fig. 5d).

Furthermore, we measured the CREB-BDNF pathway^35,36^, which is highly associated with depression. However, there were no significant differences (Fig. S3). It showed that CD36 did not exert an antidepressant effect through the neurotrophic factor pathway.

## Discussion

CD36 is an archetypal pattern-recognition receptor that binds poly-anionic ligands of both pathogen and self-origin. In the present study, we found that the expression of CD36 was significantly higher in CSDS mice and depressed patients. We also observed that, compared with WT mice, CD36^−/−^ mice displayed a significant decrease in the activation of NLRP3, the maturation of IL-1β and caspase-1. These results indicate that CD36 deficiency alters the cecal microbiome and attenuates the activation of the NLRP3 inflammasome. Our findings strengthen the potential of CD36 as a therapeutic target for modulating inflammasome-mediated pathways and the microbiome in psychiatric disorders.

We conducted approaches to investigate the critical role of CD36 in the development of depression. Our previous studies of depression have shown that molecular dysfunction occurs in the hippocampus^37,38^, PFC^27,39^, and cerebellum^40^ in animal model of depression. However, in this study, CD36 was only upregulated in the hippocampus of CSDS-exposed mice, but not in the PFC and cerebellum. Previous studies revealed that CD36 is involved in oxLDLs-related inflammation^22,23^ and lipid transport^21^, simultaneously, the lipid metabolism and inflammasome pathway were also significantly altered in the hippocampus in depression^41–44^. The results of this study may imply that CD36 has a brain-specific role in depression and acts through the lipid and inflammation pathway of the hippocampus. Moreover, CD36 was upregulated in PBMCs in depressed patients, which meant that CD36 might also exert roles in peripheral inflammatory cells.

The behavioral results of CD36^−/−^ mice demonstrated that CD36^−/−^ mice were resilient to chronic stress. CD36^−/−^ mice exposed to CSDS showed increased SI and decreased social avoidance compared with WT mice. Similar results were found in the SPT and FST, which were used to assess anhedonia and behavioral despair in rodents. The WT mice exposed to CSDS displayed reduced exploratory behavior and increased anxiety-like behavior, which were not found in CD36^−/−^ mice.

Our findings show that the gut microbiota composition of CD36^−/−^ mice is significantly altered compared with WT mice. Notably, gut microbial communities in male mice were altered shortly after exposure to social stress known to elicit anxiety-like and depressive-like behaviors^45,46^. Alpha-diversity was measured using Simpson entropy, a quantitative index that accounts for the abundance and evenness of species residing in the host, as opposed to species richness, which is the number of species present. We found that the microbial composition in CD36^−/−^ mice was less rich, but more diverse, than that in WT mice. Generally speaking, greater bacterial diversity is potentially beneficial to human health^47^. However, we previously found that there was no difference in gut microbiota alpha-diversity between depressed individuals and healthy control subjects^6^. The impact of bacterial diversity on the CNS remains controversial. Although there are many studies on the alpha-diversity of the gut microbiome in depression, its role in the disorder remains unclear.

In our current study, CD36^−/−^ mice showed marked changes in the microbiome, including upregulation of the phylum Tenericutes to the family Mycoplasmataceae, and downregulation of the class Erysipelotrichia to the genus Allobaculum (Fig. 3a). Members in the phylum Tenericutes, consisting of the sole class Mollicutes, are wall-less bacteria of several species that are among those with the smallest known genomes^48^. Defeated animals have been shown to have a decrease in Mollicutes^49^. Erysipelotrichia has been associated with nonalcoholic steatohepatitis, the pathophysiology of which involves the NLRP3 and NLRP6 inflammasomes. Allobaculum has been shown to be positively correlated with intestinal inflammation^50^. Low abundances of Allobaculum have been detected in prebiotic-fed mice^51^. Together with our findings on the changes in the inflammasome pathway in the hippocampus, these observations suggest that CD36 may modulate depressive behavior via intestinal inflammation and gut microbiota.

Three genera in the phylum bacteroidetes and five genera in the phylum firmicutes were altered in CD36^−/−^ mice. Bacteroides, rikenella, and alloprevotella, the three identified Bacteroidetes genera, were more abundant in CD36^−/−^ mice. The two most prominent phyla in the healthy microbiome are firmicutes and bacteroides, accounting for at least 70–75% of the microbiome. Decreased bacteroides is demonstrated to be associated with depression^52^. Previous animal and human studies show that bacteroides are significantly decreased in MDD patients^53,54^. Additionally, rats stressed in adulthood exhibit low rikenella abundance with low basal corticosterone levels^55^. At the genus level, the relative abundance of alloprevotella is decreased in stressed mice, and this change can be reversed by treatment with prebiotics^51^. Furthermore, the COG and KEGG pathway analysis suggest enhanced metabolic function of the cecal microbiota in CD36^−/−^ mice. This, together with the enrichment of bacteroides, rikenella, and alloprevotella, may underlie the resilience of CD36^−/−^ mice to stress-induced behavioral change.

The Ruminococcaceae family plays an important role in the maintenance of gut health by degrading cellulose and hemicellulose components from plant material. These compounds are fermented and converted into short-chain fatty acids (SCFAs), which are absorbed by the host, and are important for metabolic and immunological homeostasis. Various species of Ruminococcaceae in mice were reported to be correlated with behavioral changes induced by stress^56^. In contrast to the previous study, our findings suggest that CD36 deficiency may influence fecal SCFAs levels as well.

We found that the behavioral phenotype of CD36^−/−^ mice was also associated with disturbances in cecal microbiota. Significantly, the altered bacterial families showed a high correlation with a range of behavioral biomarkers. Along with increased SI ratio, the relative abundances of the families Brachyspiraceae and Peptostreptococcaceae were significantly increased. We also found that Lachnospiraceae was increased in tandem with the increase in sucrose preference. There is evidence to confirm that the family Brachyspiraceae and Peptostreptococcaceae are associated with intestinal bacterial infection^57,58^. Fecal specimens from mothers exposed to intimate partner violence have lower proportions of Peptostreptococcaceae at birth^59^. This finding provides insight into the involvement of the gut bacteria linking maternal psychological adversity and the maturing infant brain. Another family, Lachnospiraceae, has been observed to be associated with major depressive disorder, obesity and T2D-related phenotypes^60^. We found here that the relative abundance of Lachnospiraceae was correlated with sucrose preference. Consistent with this, previous studies show that abundance of the Lachnospiraceae family is decreased in patients with depression^61^. Lachnospiraceae participate in the breakdown of carbohydrates into SCFAs^62^, which have anti-inflammatory properties. Indeed, they have been shown to ameliorate inflammatory bowel disease, although their mechanism of action is still not completely clear^63^. These observations suggest that Lachnospiraceae may contribute to the reduced inflammation in the mice lacking CD36. Importantly, these findings support our hypothesis that CD36 affects depressive-like behavior by modulating gut microbiota and inflammation.

CD36 mediates sterile inflammation through the assembly of a Toll-like receptor 4 and 6 heterodimer^64^. Moreover, CD36^−/−^ mice show reduced secretion of NLRP3 and IL-1β^24^. Consistent with these observations, our results show that the levels of NLRP3 mRNA and protein—major contributors to caspase-1 activation and chronic stress-induced depression^10,65^—are decreased in the hippocampus of CD36^−/−^ mice. Further, ASC mRNA and protein were altered in CD36^−/−^ mice, indicating that ASC may be required for CD36-mediated regulation of NLRP3 activation and depression. Notably, full activation of the NLRP3 inflammasome requires two steps—the induction of NLRP3 and cleaved-IL-1β via transcriptional upregulation by NF-κB, and the subsequent assembly of the NLRP3 inflammasome components into a complex that activates caspase-1. In the present study, CD36 deficiency markedly decreased the activation of NF-кB, which was accompanied by decreased IL-1β secretion and caspase-1. Thus, CD36 may regulate the behavioral response to CSDS via the NLRP3 inflammasome and IL-1β pathways.

Gut microbiota have been shown to mediate systemic chronic inflammation in various diseases. Changes in ghrelin and corticotropin-releasing hormone, associated with increased gut motility^66,67^, may change microbiome composition, in turn impacting inflammation and the development of depression. Caspase-1^−/−^ mice display depressive-like behavior and anorexia together with altered gut microbiota composition after peripheral LPS administration^68,69^. Changes in inflammation are the dominant mechanisms by which the gut microbiome influences mood and stress responses, involving linkages between gut epithelial permeability and hippocampal inflammasome activation. Our findings of decrease hippocampal NLRP3 inflammasome activation and increased gut microbiome production of SCFAs, suggest that CD36 deficiency may ameliorate the depressive phenotype via anti-inflammatory effects. Further study is needed to characterize the inflammasome changes in other brain regions associated with depression, such as the prefrontal cortex.

There are several limitations of our study that should be acknowledged. First, the expression of CD36 was only verified in limited brain regions in this study. A systematic network study with several other brain regions would better elucidate the mechanism of CD36 in depression. Second, CD36 may have multiple downstream cascades. A study of transcriptomics approach is required for better systemically clarify the other mechanism of CD36. Finally, further studies of the inhibitors of NLRP3 inflammasome, CD36^−/−^ mice fecal microbiota transplantation, and depletion of CD36^−/−^ mice gut microbiota with an antibiotic cocktail could further clarify the mechanism of CD36.

## Conclusion

Our findings show that CD36 deficiency ameliorates the stress response by modulating the interaction between the inflammasome and the gut microbiota. We found a reduction of inflammasome activation in the hippocampus of CD36^−/−^ mice, accompanied by an improvement in cecal microbiota. Our findings provide a novel framework for understanding the function of CD36 through the NLRP3 inflammasome and gut microbiota, which may lead to new diagnostic and treatment strategies. Taken together, our results indicate that CD36 is critical for the development of depression-like behaviors, and that it may therefore be a potential novel therapeutic target for the treatment of depressive disorder.

## Supplementary information

Supplementary 1

Supplementary 2
