# Supplementary material for: CD36 deficiency affects depressive-like behaviors possibly by modifying gut microbiota and the inflammasome pathway in mice
Source: Transl Psychiatry. 2021 Jan 5;11:16. doi: 10.1038/s41398-020-01130-8 (PMC7791141; doi:10.1038/s41398-020-01130-8)
Supplement: Supplementary file 1 — Supplementary 1 [file 41398_2020_1130_MOESM1_ESM.docx]

**Supplementary information**

**Contents**

**Table S1. Demographic and Clinical Details of Recruited Subjects**

**Table S2. Primer pairs for RT-qPCR**

**Table S3. Discriminatory phylum between CD36^−/−^ mice and WT mice**

**Table S4. Discriminatory family between CD36^−/−^ mice and WT mice**

**Table S5. Discriminatory genus between CD36^−/−^ mice and WT mice**

**Fig. S1. CD36^−/−^ mice prevented CSDS-induced increase in immobility time in FST and decreased total distance traveled in OPT but showed no difference in the Y-maze.**

**Fig. S2. Gut microbial composition differences between CD36^−/−^ mice from WT mice.**

**Fig. S3. Representative western blotting for hippocampal CREB, TrkB, BDNF, in CD36^−/−^ mice (n = 6 mice/group).**

**Table S1. Demographic and Clinical Details of Recruited Subjects^a^**

|  | HC | MDD | P ***^b^*** |
| --- | --- | --- | --- |
| Sample Size | 31 | 24 | – |
| Medication(Y/N) | N | N | – |
| Sex (M/F) | 15/16 | 12/12 | 0.87 |
| Age (year) *^c^* | 30.3±1.6 | 33.2±1.5 | 0.198 |
| HDRS Scores *^c^* | 0.3±0.7 | 30.6±0.7 | 0.00 |

*^a^*Abbreviations: HC, Healthy controls; MDD, major depressive disorder; Y/N, yes/no; M/F, male/female;HDRS,Hamilton Depression Rating Scal

*^b^* Two-tailed Student’s *t*-test for age and HDRS. Chi-square analyses for sex.

*^c^* Values expressed as the means ± standard deviations.

**Table S2. Primer pairs for RT-qPCR**

| **Table 2. Primer pairs for RT-qPCR** | | |  |
| --- | --- | --- | --- |
| Gene | | Forwar Primer (5’to 3’) | Reverse Primer (5’to 3’) |
| CD36 | | GAACCACTGCTTTCAAAAACTGG | TGCTGTTCTTTGCCACGTCA |
| NLRP3 | | GTGGTGACCCTCTGTGAGGT | TCTTCCTGGAGCGCTTCTAA |
| ASC | | GAAGCTGCTGACAGTGCAAC | GCCACAGCTCCAGACTCTTC |
| pro-Caspase-1 | | AGA TGG CAC ATT TCC AGG AC | GAT CCT CCA GCA GCA ACT TC |
| Caspase-1 | | ACAAGGCACGGGACCTATG | TCCCAGTCAGTCCTGGAAATG |
| pro- IL-1β | | TCT TTG AAGTTGACG GACCC | TGA GTGATACTGCCTGCCTG |
| IL-1β | | GGTCAAAGGTTTGGAAGCAG | TGTGAAATGCCACCTTTTGA |
| β-Actin | | GCCACCAGTTCGCCATGGAT | TCTGGGCCTCGTCACCCACATA |

**Table S3. Discriminatory phylum between CD36^−/−^ mice and WT mice**

| **Species name** | **Relative Abundance in CD36^−/−^ mice (%)** | | **Relative Abundance in WT mice (%)** | | **p-value** | **CD36^−/−^ mice Relative to WT mice** |
| --- | --- | --- | --- | --- | --- | --- |
|  | **Mean** | **Std. Deviation** | **Mean** | **Std. Deviation** |  |  |
| p__Tenericutes | 0.9983 | 0.6284 | 0.2218 | 0.1064 | 0.002492 | Up |
| p__Spirochaetae | 0.0229 | 0.03639 | 3.964 | 7.289 | 0.1777 | Down |
| p__Cyanobacteria | 0.1287 | 0.1825 | 0.04396 | 0.05281 | 0.2031 | Up |
| p__Saccharibacteria | 0.1167 | 0.08646 | 0.2304 | 0.2369 | 0.2496 | Down |
| p__Firmicutes | 53.75 | 11.39 | 47.13 | 13.95 | 0.3264 | Up |
| p__Proteobacteria | 4.903 | 3.811 | 3.708 | 1.439 | 0.3984 | Up |
| p__Bacteroidetes | 37.4 | 10.39 | 41.83 | 11.78 | 0.4456 | Down |
| p__Actinobacteria | 0.7148 | 1.08 | 0.7961 | 0.5705 | 0.8484 | Down |
| p__Deferribacteres | 0.4309 | 0.4523 | 0.4128 | 0.2825 | 0.9231 | Up |
| p__Verrucomicrobia | 1.537 | 3.701 | 1.661 | 2.908 | 0.9412 | Down |

**Table S4. Discriminatory family between CD36^−/−^ mice and WT mice**

| **Species name** | **Relative Abundance in CD36^−/−^ mice (%)** | | **Relative Abundance in WT mice (%)** | | **Pvalue** | **CD36^−/−^ mice Relative to WT mice** |
| --- | --- | --- | --- | --- | --- | --- |
|  | **Mean** | **Std. Deviation** | **Mean** | **Std. Deviation** |  |  |
| f__Mycoplasmataceae | 0.7542 | 0.5081 | 0.01232 | 0.01691 | 0.000579 | Up |
| f__Bacteroidaceae | 1.278 | 0.4707 | 0.4596 | 0.2944 | 0.000775 | Up |
| f__Erysipelotrichaceae | 0.7504 | 0.6095 | 2.595 | 1.683 | 0.01581 | Down |
| f__Rikenellaceae | 0.9131 | 0.5172 | 0.4023 | 0.2346 | 0.01896 | Up |
| f__Ruminococcaceae | 13.98 | 5.304 | 8.365 | 3.313 | 0.02097 | Up |
| f__Christensenellaceae | 0.002724 | 0.005021 | 0.01366 | 0.01046 | 0.02386 | Down |
| f__Peptococcaceae | 0.2819 | 0.1096 | 0.118 | 0.1974 | 0.06958 | Up |
| f__Unclassified_o__Bacteroidales | 0.000425 | 0.001125 | 0.02302 | 0.03269 | 0.09122 | Down |
| f__Bacteroidales_S24-7_group | 22.32 | 8.433 | 31.64 | 11.37 | 0.09167 | Down |
| f__Brachyspiraceae | 0.01511 | 0.02559 | 0.000405 | 0.001216 | 0.104 | Up |
| f__Unclassified_p__Bacteroidetes | 0 | 0 | 0.007662 | 0.01172 | 0.1082 | Down |
| f__Succinivibrionaceae | 0 | 0 | 0.001451 | 0.00228 | 0.1171 | Down |
| f__Veillonellaceae | 0.1037 | 0.2646 | 0.4716 | 0.587 | 0.1477 | Down |
| f__Staphylococcaceae | 0.01942 | 0.03086 | 0.1637 | 0.2543 | 0.1606 | Down |
| f__Family_XIII | 0.1611 | 0.08904 | 0.1112 | 0.04658 | 0.1676 | Up |
| f__Spirochaetaceae | 0.00779 | 0.02061 | 3.963 | 7.288 | 0.1761 | Down |
| f__norank_o__Gastranaerophilales | 0.1283 | 0.1814 | 0.04352 | 0.05316 | 0.2011 | Up |
| f__Defluviitaleaceae | 0 | 0 | 0.000794 | 0.001587 | 0.2105 | Down |
| f__norank_o__Mollicutes_RF9 | 0.09009 | 0.09872 | 0.1545 | 0.1052 | 0.2326 | Down |
| f__Anaeroplasmataceae | 0.1541 | 0.2102 | 0.05491 | 0.1113 | 0.2426 | Up |
| f__Unknown_Family | 0.1167 | 0.08646 | 0.2304 | 0.2369 | 0.2496 | Down |
| f__Peptostreptococcaceae | 0.008193 | 0.02168 | 0 | 0 | 0.2711 | Down |
| f__Aerococcaceae | 0.000425 | 0.001125 | 0.003449 | 0.006878 | 0.2723 | Down |
| f__Bifidobacteriaceae | 0.01948 | 0.0231 | 0.03972 | 0.04333 | 0.2844 | Down |
| f__Clostridiaceae_1 | 0.1688 | 0.2865 | 0.0616 | 0.07788 | 0.2977 | Up |
| f__Streptococcaceae | 0.03026 | 0.02527 | 0.04485 | 0.02922 | 0.312 | Down |
| f__Unclassified_p__Firmicutes | 0 | 0 | 0.00547 | 0.01374 | 0.3136 | Down |
| f__Corynebacteriaceae | 0.002477 | 0.004625 | 0.02126 | 0.04773 | 0.3206 | Down |
| f__Dermabacteraceae | 0.000393 | 0.001039 | 0.005494 | 0.01317 | 0.3277 | Down |
| f__Propionibacteriaceae | 0.001562 | 0.001466 | 0.000671 | 0.002011 | 0.3421 | Up |
| f__Unclassified_o__Clostridiales | 0.03288 | 0.04256 | 0.01822 | 0.02293 | 0.3902 | Up |
| f__Planococcaceae | 0 | 0 | 0.003528 | 0.01058 | 0.3963 | Down |
| f__Desulfovibrionaceae | 2.761 | 3.172 | 1.758 | 1.24 | 0.3971 | Up |
| f__Enterobacteriaceae | 0.005072 | 0.009182 | 0.01051 | 0.0166 | 0.4512 | Down |
| f__Moraxellaceae | 0.02466 | 0.06525 | 0.007894 | 0.02368 | 0.4843 | Up |
| f__Prevotellaceae | 12.43 | 12.11 | 8.861 | 10.17 | 0.5318 | Up |
| f__Lachnospiraceae | 36.4 | 10.87 | 32.83 | 11.96 | 0.5474 | Up |
| f__Bacillaceae | 0.06159 | 0.02533 | 0.04974 | 0.04587 | 0.5507 | Up |
| f__Lactobacillaceae | 1.662 | 1.788 | 2.187 | 2.756 | 0.6697 | Down |
| f__Pseudomonadaceae | 0.002248 | 0.003167 | 0.003474 | 0.009187 | 0.7421 | Up |
| f__Enterococcaceae | 0.002762 | 0.002773 | 0.002315 | 0.002657 | 0.748 | Up |
| f__Alcaligenaceae | 1.02 | 0.709 | 0.9404 | 0.7003 | 0.8262 | Up |
| f__Rhodospirillaceae | 0.01186 | 0.01502 | 0.01053 | 0.009202 | 0.8293 | Up |
| f__Helicobacteraceae | 1.079 | 1.459 | 0.9763 | 1.115 | 0.8755 | Up |
| f__Porphyromonadaceae | 0.4615 | 0.2734 | 0.4389 | 0.3732 | 0.8949 | Up |
| f__Deferribacteraceae | 0.4309 | 0.4523 | 0.4128 | 0.2825 | 0.9231 | Up |
| f__Coriobacteriaceae | 0.6909 | 1.066 | 0.7289 | 0.5745 | 0.9281 | Down |
| f__Verrucomicrobiaceae | 1.537 | 3.701 | 1.661 | 2.908 | 0.9412 | Down |
| f__Clostridiales_vadinBB60_group | 0.08662 | 0.05258 | 0.08861 | 0.08434 | 0.9575 | Down |
| f__norank_c__Cyanobacteria | 0.000455 | 0.001204 | 0.000441 | 0.001323 | 0.9827 | Up |

**Table S5. Discriminatory genus between CD36^−/−^ mice and WT mice**

| **Species name** | **Relative Abundance in CD36^−/−^ mice (%)** | | **Relative Abundance in WT mice (%)** | | **Pvalue** | **CD36^−/−^ mice Relative to WT mice** |
| --- | --- | --- | --- | --- | --- | --- |
|  | **Mean** | **Std. Deviation** | **Mean** | **Std. Deviation** |  |  |
| g__uncultured_f__Mycoplasmataceae | 0.7301 | 0.4919 | 0 | 0 | 0.000501 | Up |
| g__Bacteroides | 1.278 | 0.4707 | 0.4596 | 0.2944 | 0.000775 | Up |
| g__Unclassified_f__Ruminococcaceae | 0.959 | 0.5278 | 0.3541 | 0.2029 | 0.006748 | Up |
| g__Tyzzerella_3 | 0.03936 | 0.04513 | 3.839 | 3.393 | 0.01076 | Down |
| g__Ruminococcaceae_UCG-013 | 0.1522 | 0.1536 | 0.003859 | 0.004863 | 0.01104 | Up |
| g__Allobaculum | 0.6014 | 0.6412 | 2.456 | 1.576 | 0.01134 | Down |
| g__Rikenella | 0.3639 | 0.3397 | 0.05243 | 0.06521 | 0.01682 | Up |
| g__Christensenellaceae_R-7_group | 0.002353 | 0.005127 | 0.0133 | 0.01067 | 0.02607 | Down |
| g__Alloprevotella | 0.6479 | 0.4311 | 0.161 | 0.355 | 0.02641 | Up |
| g__Roseburia | 0.2333 | 0.3356 | 1.667 | 1.49 | 0.02649 | Down |
| g__Ruminococcaceae_UCG-010 | 0.07118 | 0.0357 | 0.03767 | 0.02915 | 0.05747 | Up |
| g__Lachnospiraceae_NK4A136_group | 24.51 | 11 | 15.62 | 6.248 | 0.05981 | Up |
| g__[Eubacterium]_brachy_group | 0.04607 | 0.04424 | 0.01611 | 0.01377 | 0.07395 | Up |
| g__[Eubacterium]_nodatum_group | 0.04445 | 0.03982 | 0.01707 | 0.01404 | 0.07413 | Up |
| g__Alistipes | 0.4472 | 0.1663 | 0.2875 | 0.1746 | 0.08526 | Up |
| g__Unclassified_o__Bacteroidales | 0.000425 | 0.001125 | 0.02302 | 0.03269 | 0.09122 | Down |
| g__norank_f__Bacteroidales_S24-7_group | 22.32 | 8.433 | 31.64 | 11.37 | 0.09167 | Down |
| g__Unclassified_f__Erysipelotrichaceae | 0.000371 | 0.000981 | 0.007163 | 0.009853 | 0.09298 | Down |
| g__Ruminococcus_1 | 0.5457 | 0.3309 | 0.2983 | 0.2291 | 0.0985 | Up |
| g__Ruminiclostridium_6 | 0.9147 | 0.9763 | 0.3228 | 0.2858 | 0.1036 | Up |
| g__Brachyspira | 0.01511 | 0.02559 | 0.000405 | 0.001216 | 0.104 | Up |
| g__Unclassified_p__Bacteroidetes | 0 | 0 | 0.007662 | 0.01172 | 0.1082 | Down |
| g__Anaerobiospirillum | 0 | 0 | 0.001451 | 0.00228 | 0.1171 | Down |
| g__Bilophila | 0.02071 | 0.02428 | 0.006189 | 0.009105 | 0.1184 | Up |
| g__uncultured_f__Peptococcaceae | 0.2265 | 0.1249 | 0.09037 | 0.1928 | 0.1282 | Up |
| g__Peptococcus | 0.0554 | 0.04627 | 0.0276 | 0.0222 | 0.1335 | Up |
| g__uncultured_f__Ruminococcaceae | 1.078 | 0.4053 | 0.6809 | 0.576 | 0.1448 | Up |
| g__Unclassified_f__Veillonellaceae | 0.1037 | 0.2646 | 0.4716 | 0.587 | 0.1477 | Down |
| g__Staphylococcus | 0.01769 | 0.02796 | 0.1593 | 0.2473 | 0.1568 | Down |
| g__uncultured_f__Lachnospiraceae | 4.725 | 0.9817 | 3.73 | 1.541 | 0.16 | Up |
| g__Ruminiclostridium_5 | 6.75 | 5.076 | 3.903 | 2.64 | 0.167 | Up |
| g__Ruminiclostridium_9 | 1.053 | 0.4367 | 0.7503 | 0.4004 | 0.1717 | Up |
| g__Treponema_2 | 0.00779 | 0.02061 | 3.963 | 7.288 | 0.1761 | Down |
| g__uncultured_f__Erysipelotrichaceae | 0.09061 | 0.07394 | 0.04251 | 0.06195 | 0.1783 | Up |
| g__Lachnospiraceae_UCG-006 | 0.2972 | 0.2234 | 0.1814 | 0.098 | 0.1829 | Up |
| g__Parabacteroides | 0.2609 | 0.143 | 0.1756 | 0.1041 | 0.1883 | Up |
| g__Marvinbryantia | 0.01005 | 0.01513 | 0.002302 | 0.006907 | 0.1913 | Up |
| g__Lachnospiraceae_UCG-008 | 0.01748 | 0.02896 | 0.004145 | 0.00546 | 0.1944 | Up |
| g__norank_o__Gastranaerophilales | 0.1283 | 0.1814 | 0.04352 | 0.05316 | 0.2011 | Up |
| g__Defluviitaleaceae_UCG-011 | 0 | 0 | 0.000794 | 0.001587 | 0.2105 | Down |
| g__norank_o__Mollicutes_RF9 | 0.09009 | 0.09872 | 0.1545 | 0.1052 | 0.2326 | Down |
| g__Clostridium_sensu_stricto_1 | 0.0732 | 0.1765 | 0.00186 | 0.004382 | 0.2409 | Up |
| g__Anaeroplasma | 0.1541 | 0.2102 | 0.05491 | 0.1113 | 0.2426 | Up |
| g__Unclassified_f__Prevotellaceae | 0 | 0 | 0.05836 | 0.1258 | 0.2433 | Down |
| g__Candidatus_Saccharimonas | 0.1167 | 0.08646 | 0.2304 | 0.2369 | 0.2496 | Down |
| g__Lachnospiraceae_UCG-010 | 0.00412 | 0.003293 | 0.01056 | 0.01421 | 0.2629 | Down |
| g__Romboutsia | 0.008193 | 0.02168 | 0 | 0 | 0.2711 | Up |
| g__Facklamia | 0.000425 | 0.001125 | 0.003449 | 0.006878 | 0.2723 | Down |
| g__Incertae_Sedis_f__Lachnospiraceae | 0.523 | 0.8694 | 0.9425 | 0.6047 | 0.2732 | Down |
| g__Acetatifactor | 0.1547 | 0.09416 | 0.09098 | 0.1234 | 0.2774 | Up |
| g__[Eubacterium]_ventriosum_group | 0 | 0 | 0.005538 | 0.01287 | 0.2775 | Down |
| g__Bifidobacterium | 0.01948 | 0.0231 | 0.03972 | 0.04333 | 0.2844 | Down |
| g__Ruminococcaceae_UCG-005 | 0.03618 | 0.02858 | 0.02251 | 0.02074 | 0.2852 | Up |
| g__Ruminiclostridium | 0.3323 | 0.1204 | 0.2442 | 0.1806 | 0.2864 | Up |
| g__Unclassified_p__Firmicutes | 0 | 0 | 0.00547 | 0.01374 | 0.3136 | Down |
| g__Corynebacterium_1 | 0.002477 | 0.004625 | 0.02126 | 0.04773 | 0.3206 | Down |
| g__Lactococcus | 0.0254 | 0.02406 | 0.03975 | 0.03068 | 0.3272 | Down |
| g__Ruminococcaceae_UCG-004 | 0.08405 | 0.07398 | 0.1246 | 0.083 | 0.3274 | Down |
| g__Brachybacterium | 0.000393 | 0.001039 | 0.005494 | 0.01317 | 0.3277 | Down |
| g__Propionibacterium | 0.001562 | 0.001466 | 0.000671 | 0.002011 | 0.3421 | Up |
| g__Ureaplasma | 0.01582 | 0.02095 | 0.008035 | 0.0119 | 0.3624 | Up |
| g__Parvibacter | 0.005343 | 0.00673 | 0.002611 | 0.005385 | 0.3816 | Up |
| g__Jeotgalicoccus | 0.001731 | 0.003389 | 0.004415 | 0.007331 | 0.3874 | Down |
| g__Lachnoclostridium | 0.5183 | 0.314 | 0.4033 | 0.2037 | 0.3892 | Up |
| g__Unclassified_o__Clostridiales | 0.03288 | 0.04256 | 0.01822 | 0.02293 | 0.3902 | Up |
| g__Lysinibacillus | 0 | 0 | 0.003528 | 0.01058 | 0.3963 | Down |
| g__Candidatus_Stoquefichus | 0 | 0 | 0.000436 | 0.001308 | 0.3963 | Down |
| g__Paraprevotella | 0.08695 | 0.06922 | 0.1634 | 0.2214 | 0.3965 | Down |
| g__Desulfovibrio | 2.74 | 3.177 | 1.751 | 1.239 | 0.4043 | Up |
| g__Rikenellaceae_RC9_gut_group | 0.1021 | 0.1265 | 0.06238 | 0.0521 | 0.4043 | Up |
| g__Gordonibacter | 0.02939 | 0.03791 | 0.05442 | 0.07008 | 0.4103 | Down |
| g__Coriobacteriaceae_UCG-002 | 0.1818 | 0.4484 | 0.05733 | 0.05594 | 0.4191 | Up |
| g__Coprococcus_1 | 0.3934 | 0.4139 | 0.2755 | 0.1239 | 0.428 | Up |
| g__Escherichia-Shigella | 0.005072 | 0.009182 | 0.01051 | 0.0166 | 0.4512 | Down |
| g__Lachnospiraceae_UCG-001 | 0.7712 | 1.253 | 1.479 | 2.239 | 0.4675 | Down |
| g__Psychrobacter | 0.02466 | 0.06525 | 0.007894 | 0.02368 | 0.4843 | Up |
| g__Candidatus_Arthromitus | 0.09558 | 0.1209 | 0.05974 | 0.07922 | 0.4854 | Up |
| g__Anaerovorax | 0.000742 | 0.001963 | 0.001492 | 0.002366 | 0.5102 | Down |
| g__Lachnospiraceae_FCS020_group | 0.04251 | 0.04093 | 0.05497 | 0.03487 | 0.5214 | Down |
| g__Erysipelatoclostridium | 0.03763 | 0.02305 | 0.04963 | 0.04634 | 0.5424 | Down |
| g__Mycoplasma | 0.008291 | 0.01708 | 0.004282 | 0.008619 | 0.5487 | Up |
| g__Bacillus | 0.06159 | 0.02533 | 0.04974 | 0.04587 | 0.5507 | Up |
| g__Anaerotruncus | 0.6452 | 0.6752 | 0.4982 | 0.2949 | 0.5649 | Up |
| g__uncultured_f__Coriobacteriaceae | 0.01307 | 0.01785 | 0.008607 | 0.01332 | 0.5752 | Up |
| g__Prevotellaceae_UCG-001 | 10.87 | 12.57 | 7.756 | 10.28 | 0.593 | Up |
| g__Unclassified_f__Coriobacteriaceae | 0.272 | 0.4553 | 0.3787 | 0.3606 | 0.6081 | Down |
| g__Ruminococcaceae_UCG-009 | 0.06266 | 0.02057 | 0.05179 | 0.05444 | 0.6262 | Up |
| g__Ruminococcaceae_UCG-014 | 0.6097 | 0.4479 | 0.4941 | 0.4714 | 0.627 | Up |
| g__Turicibacter | 0.01462 | 0.02042 | 0.03419 | 0.1026 | 0.6293 | Down |
| g__Ruminiclostridium_1 | 0.000786 | 0.002079 | 0.000405 | 0.001216 | 0.6529 | Up |
| g__Oscillibacter | 0.5491 | 0.2674 | 0.4731 | 0.3708 | 0.6551 | Up |
| g__Lactobacillus | 1.662 | 1.788 | 2.187 | 2.756 | 0.6697 | Down |
| g__[Eubacterium]_coprostanoligenes_group | 0.07067 | 0.1691 | 0.04468 | 0.0567 | 0.6705 | Up |
| g__Odoribacter | 0.2006 | 0.2192 | 0.2632 | 0.3522 | 0.6875 | Down |
| g__Family_XIII_AD3011_group | 0.008087 | 0.01033 | 0.0118 | 0.0221 | 0.6889 | Down |
| g__Enterorhabdus | 0.1337 | 0.1076 | 0.1564 | 0.1288 | 0.7144 | Down |
| g__norank_f__Erysipelotrichaceae | 0.005766 | 0.009584 | 0.004273 | 0.007228 | 0.727 | Up |
| g__Pseudomonas | 0.002248 | 0.003167 | 0.003474 | 0.009187 | 0.7421 | Down |
| g__Adlercreutzia | 0.05562 | 0.0926 | 0.07088 | 0.08884 | 0.7429 | Down |
| g__Enterococcus | 0.002762 | 0.002773 | 0.002315 | 0.002657 | 0.748 | Up |
| g__Tyzzerella | 0.0256 | 0.015 | 0.03035 | 0.03734 | 0.7568 | Down |
| g__Unclassified_f__Lachnospiraceae | 3.965 | 2.069 | 4.289 | 2.641 | 0.7937 | Down |
| g__Blautia | 0.1744 | 0.1772 | 0.203 | 0.279 | 0.817 | Down |
| g__Parasutterella | 1.02 | 0.709 | 0.9404 | 0.7003 | 0.8262 | Up |
| g__Thalassospira | 0.01186 | 0.01502 | 0.01053 | 0.009202 | 0.8293 | Up |
| g__Prevotellaceae_UCG-003 | 0.8193 | 1.047 | 0.7224 | 0.9655 | 0.8504 | Up |
| g__Family_XIII_UCG-001 | 0.0618 | 0.03473 | 0.06472 | 0.03564 | 0.8718 | Down |
| g__Helicobacter | 1.079 | 1.459 | 0.9763 | 1.115 | 0.8755 | Up |
| g__Streptococcus | 0.004857 | 0.003872 | 0.0051 | 0.00468 | 0.9131 | Down |
| g__Mucispirillum | 0.4309 | 0.4523 | 0.4128 | 0.2825 | 0.9231 | Up |
| g__Akkermansia | 1.537 | 3.701 | 1.661 | 2.908 | 0.9412 | Down |
| g__norank_f__Clostridiales_vadinBB60_group | 0.08662 | 0.05258 | 0.08861 | 0.08434 | 0.9575 | Down |
| g__uncultured_f__Christensenellaceae | 0.000371 | 0.000981 | 0.000353 | 0.001058 | 0.9725 | Up |
| g__norank_c__Cyanobacteria | 0.000455 | 0.001204 | 0.000441 | 0.001323 | 0.9827 | Up |
| g__Ruminococcaceae_NK4A214_group | 0.06112 | 0.02209 | 0.06122 | 0.01965 | 0.9923 | Down |

**
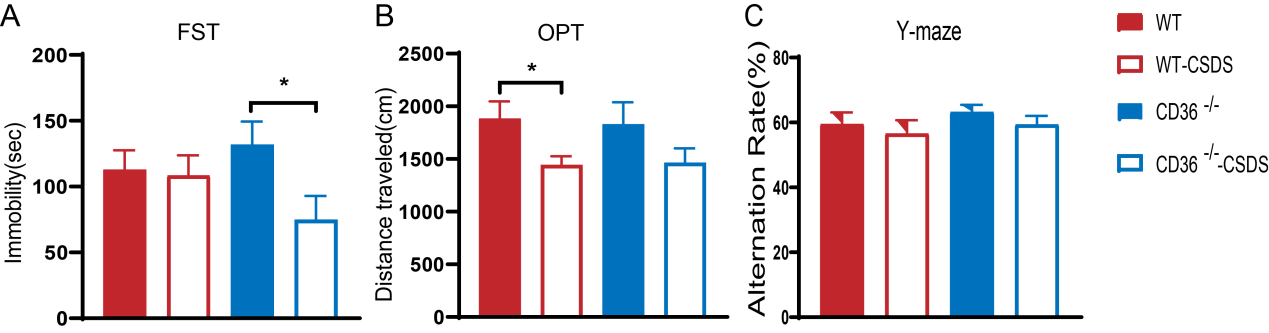
**

**Figure S1.** CD36^−/−^ mice prevented CSDS-induced increase in immobility time in FST and decreased total distance traveled in OPT but showed no difference in the Y-maze. (A) CD36^−/−^ mice prevented CSDS-induced increase in immobility time in FST. (B) WT mice showed decreased total distance traveled in OPT. (C) The alternation percentage of CD36^−/−^ mice and WT mice in the Y-maze (n=11-14 mice/group, P < 0.05).

**
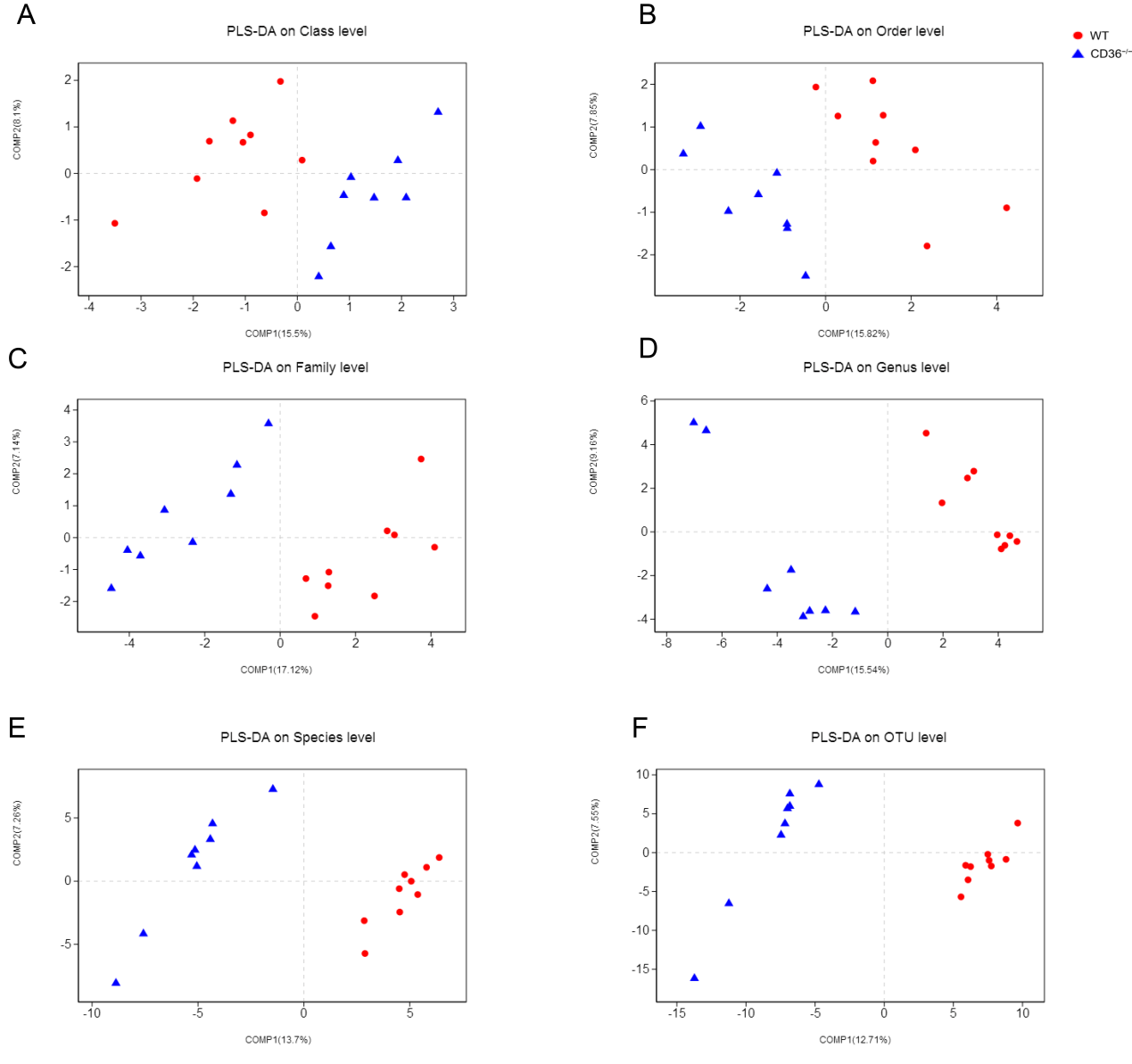
**

**Figure S2.** Gut microbial composition differences between CD36^−/−^ mice from WT mice. (A-F) The microbial composition of CD36^−/−^ mice was significantly different from that in WT mice from class to OTU levels (n=8-9 mice/group).

**
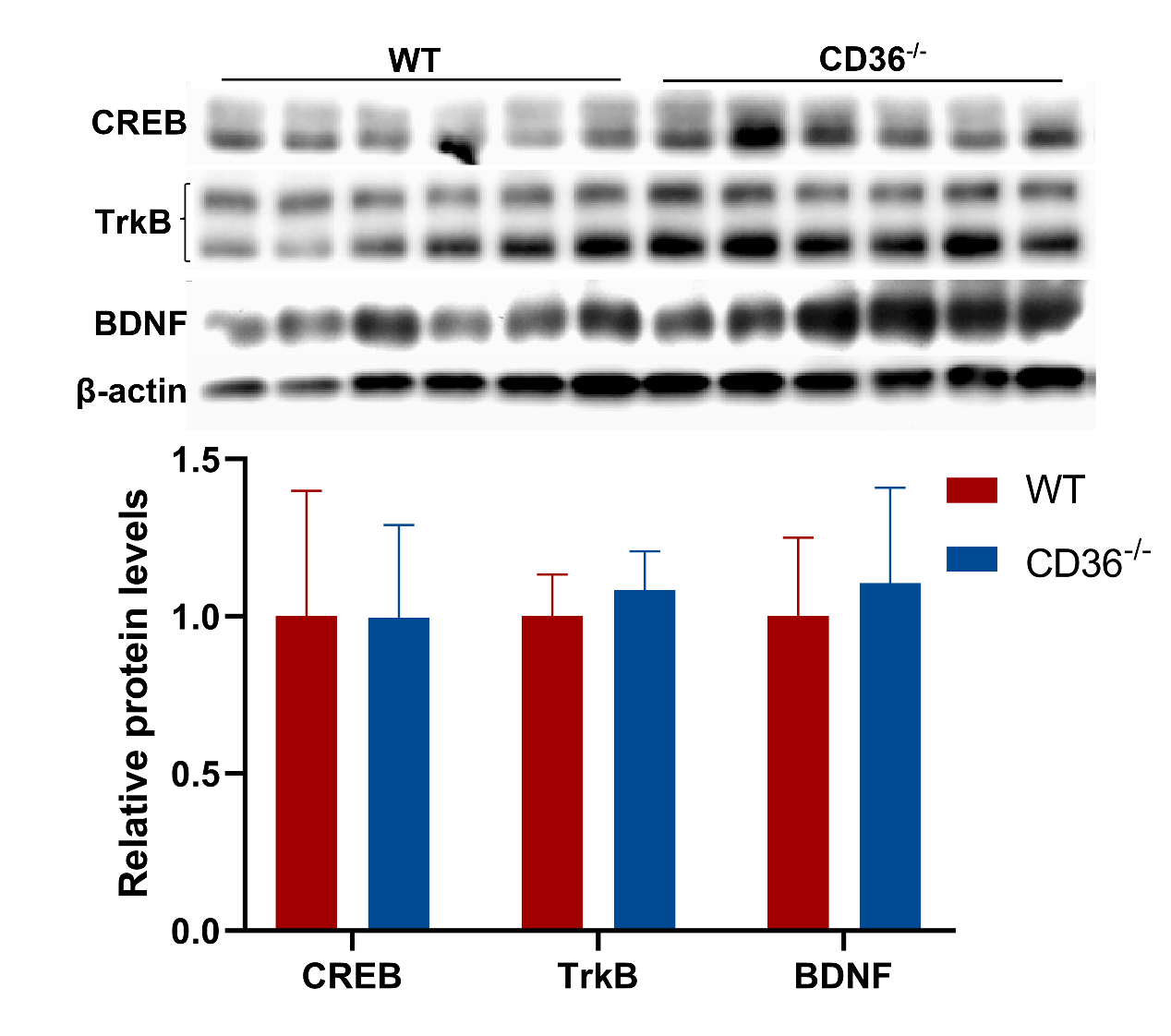
**

**Figure S3.** Representative western blotting for hippocampal CREB, TrkB, BDNF, in CD36^−/−^ mice (n = 6 mice/group).
